# Supplementary material for: Modulation of riboflavin biosynthesis and utilization in mycobacteria
Source: Microbiol Spectr. 2024 Jun 25;12(8):e03207-23. doi: 10.1128/spectrum.03207-23 (PMC11302143; doi:10.1128/spectrum.03207-23)
Supplement: Supplemental material — Fig. S1 to S11; Tables S1 to S9. [file spectrum.03207-23-s0001.pdf]

SUPPLEMENTARY INFORMATION

Modulation of riboflavin biosynthesis and utilization in mycobacteria

Melissa D. Chengalroyen[a]#, Carolina Mehaffy[b], Megan Lucas[b], Niel Bauer[b], Mabule L. Raphela[a], Nurudeen Oketade[b], Digby F. Warner[a,c], Deborah Lewinsohn[d], David Lewinsohn[d], Karen M. Dobos[b] & Valerie Mizrahi[a,c]#

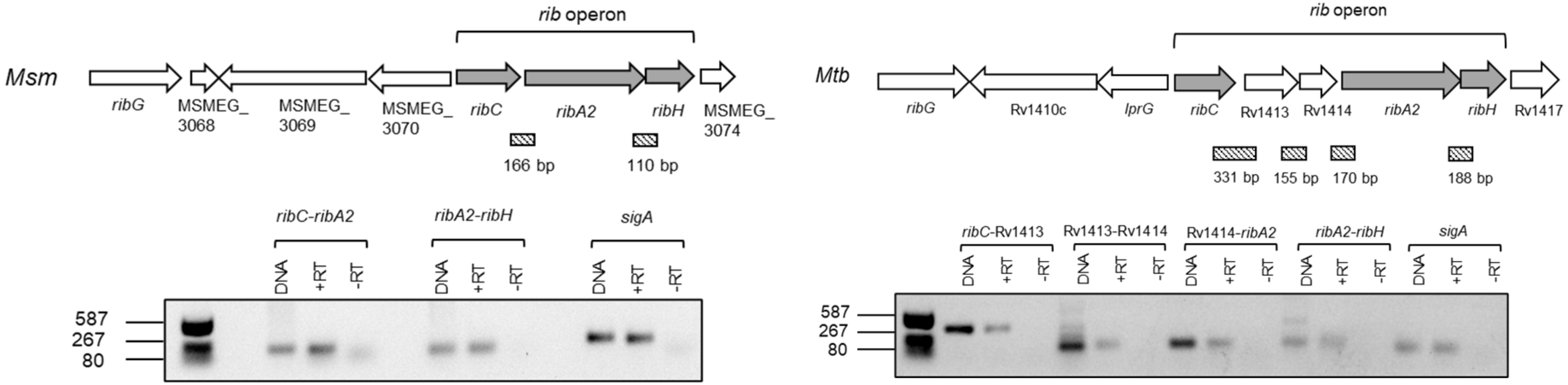

FIG. S1. RF biosynthesis (*rib*) operons in Msm and Mtb. Clustered *rib* genes are shown in grey. Also shown is an agarose gel electrophoresis of PCR products (sizes represented by diagonal line box) from cDNA template, using primers designed to amplify junctions between genes. Reactions were performed either in the presence (+RT) or absence of reverse transcriptase (-RT) and genomic DNA was used as a control.

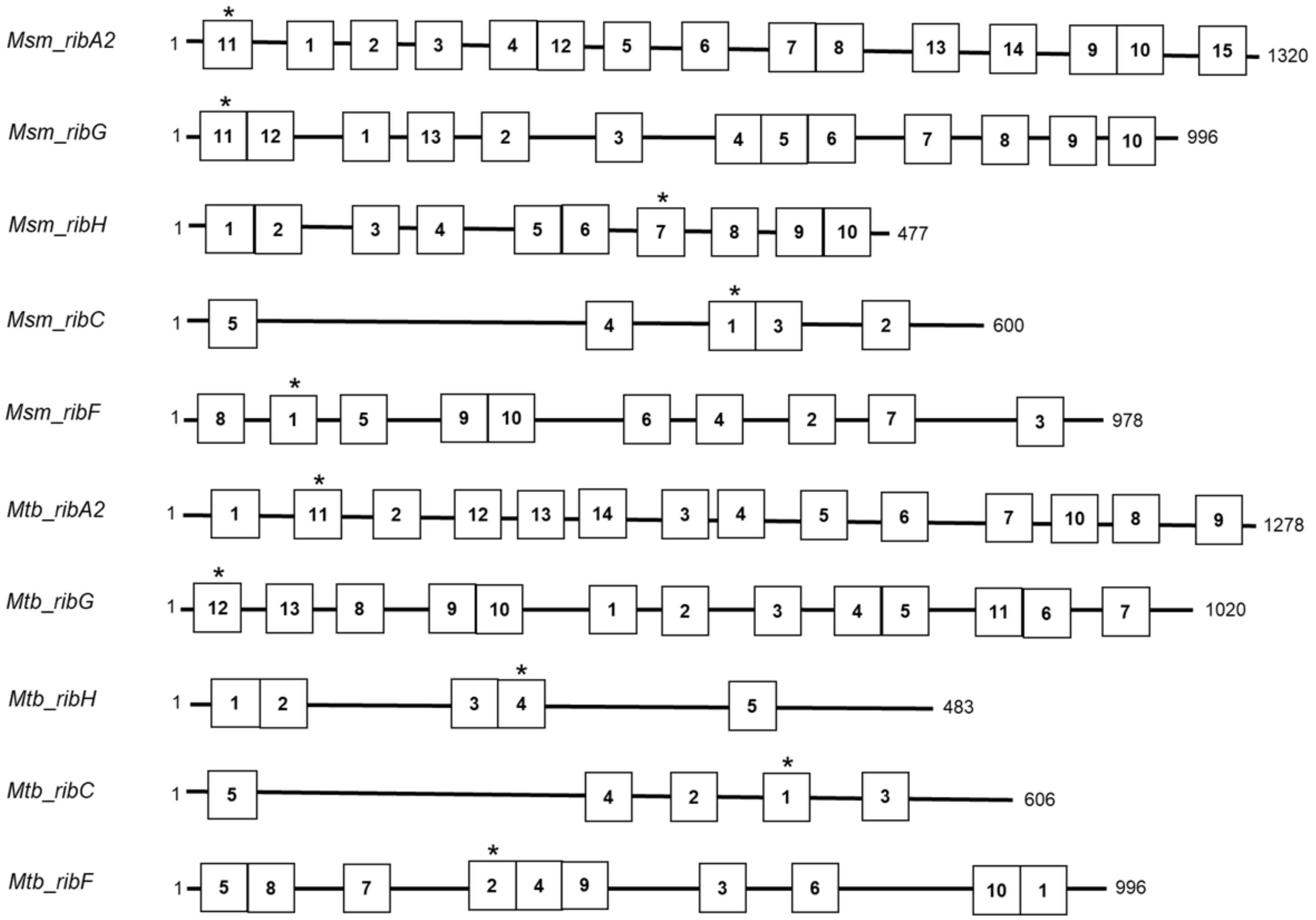

FIG. S2. Approximate positions of sgRNAs used to create hypomorphs in RF pathway genes. sgRNAs next to one another represent those sharing overlapping sequences. The sgRNA sequences are provided in Tables S1 and S2. Asterisks denote the sgRNA selected for detailed characterization. Not drawn to scale.

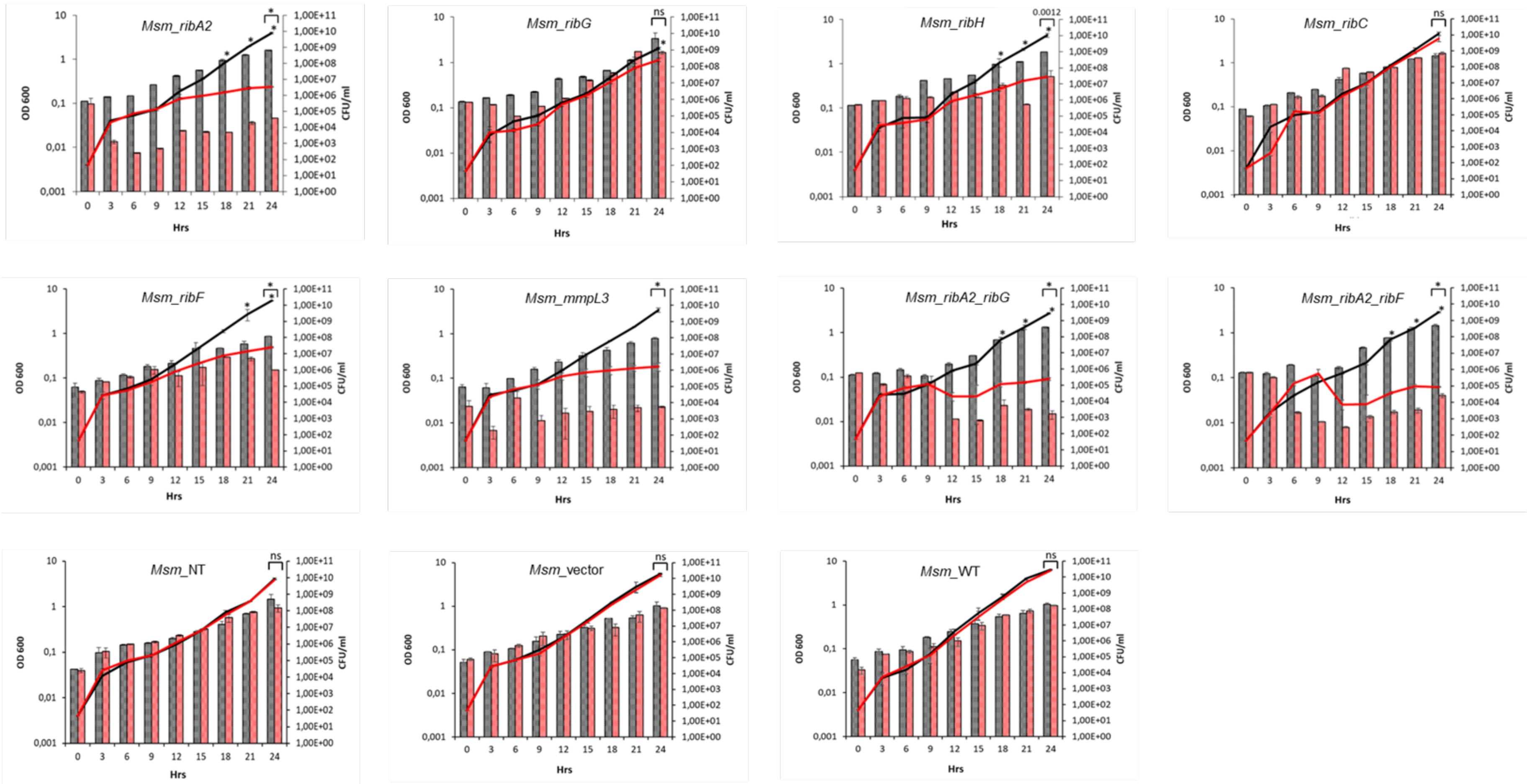

FIG. S3. Impact of RF pathway gene silencing on growth and viability of Msm in liquid culture. Cultures were monitored every 3 h by measuring OD_{600} (solid lines) and enumerating CFUs by plating serial dilutions on 7H10 agar (columns). Cultures were grown without ATc (black solid line and black column) or in the presence of 100 ng/ml ATc (red solid line and red column). Error bars represent the SD derived from two biological replicates. Statistical comparisons were performed using a two-way ANOVA and Sidak's multiple comparison test whereby statistical significance is represented by $p < 0.0001$, denoted by an asterisk and no statistical difference represented as ns. When assessing CFUs over time, the p-value is only shown for the end time point of 24 h (indicated with a square bracket). When assessing ODs over time, any significant difference in growth from 0-24 h is shown by an asterisk above the specified time point.

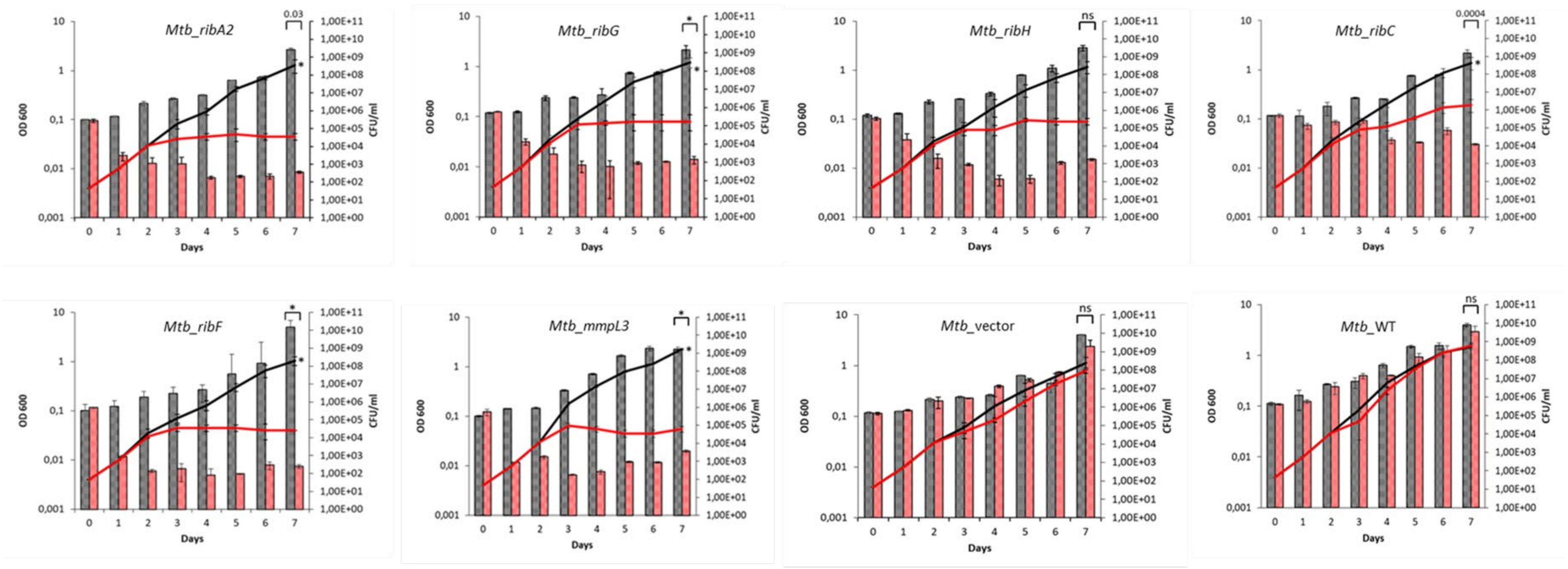

FIG. S4. Impact of RF pathway gene silencing on growth and viability of Mtb in liquid culture. Cultures were monitored every 24 h by measuring OD_{600} (solid lines) and enumerating CFUs by plating serial dilutions on 7H10 agar (columns). Cultures were grown without ATc (black solid line and black column) or in the presence of 100 ng/ml ATc (red solid line and red column). Error bars represent the SD derived from two biological replicates. Statistical comparisons were performed using a two-way ANOVA and Sidak's multiple comparison test whereby statistical significance is represented by $p < 0.0001$, denoted by an asterisk and no statistical difference represented as ns. When assessing CFUs over time, the p-value is only shown for the end time point of 7 days (indicated with a square bracket). When assessing ODs over time, any significant difference in growth from 0-7 days is shown by an asterisk above the specified time point.

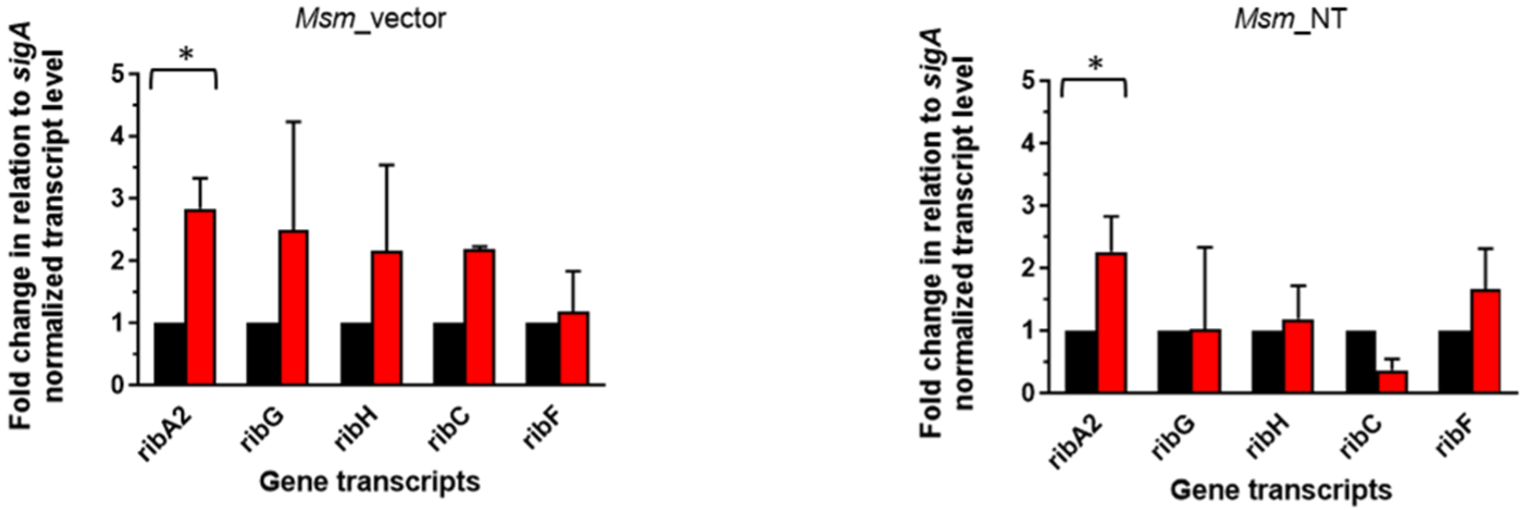

FIG. S5. Impact of the CRISPRi system on ATc responsiveness of RF pathway gene expression. Msm_vector harbours the CRISPRi vector backbone without a sgRNA and Msm_NT harbours the CRISPRi vector with a scrambled non-targeting sgRNA. Fold gene expression in the presence of ATc (red bars) is compared to the absence of ATc (black bars). Error bars represent the SD derived from three biological replicates. Statistical comparisons were performed using a two-way ANOVA and either the Sidak's or Dunnett's multiple comparison test whereby statistical significance is represented by $p < 0.05$, denoted by an asterisk.

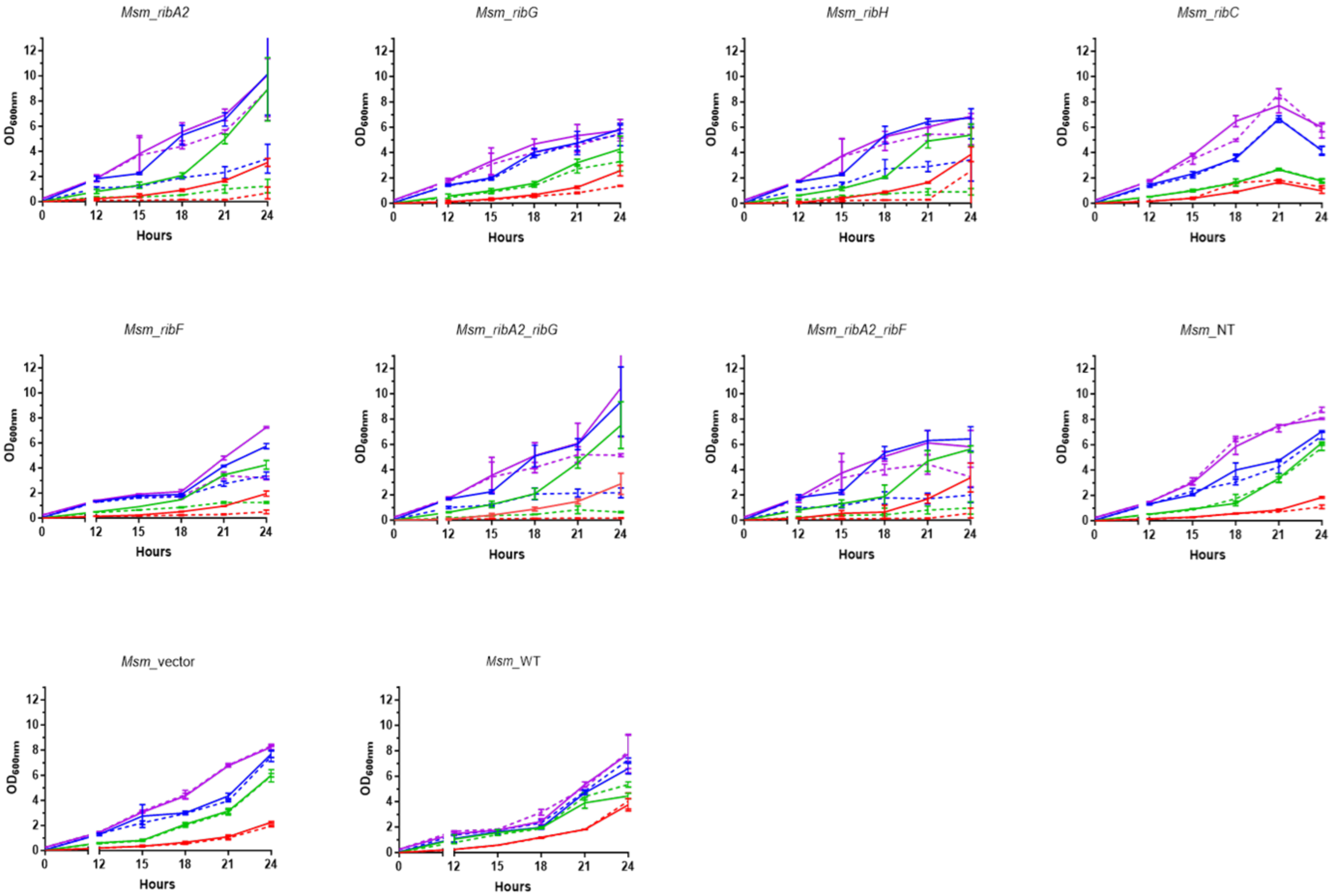

FIG. S6. Efficacy of CRISPRi-dependent growth repression of Msm hypomorphs as a function of inoculum size. Kinetics of growth of Msm hypomorph cultures inoculated with cells at an OD_{600} value of 0.004 (red), 0.016 (green), 0.06 (blue) or 0.25 (purple). The solid line represents growth in the absence of ATc and the dashed line in the presence of 100 ng/ml ATc. Error bars represent the SD derived from two biological replicates.

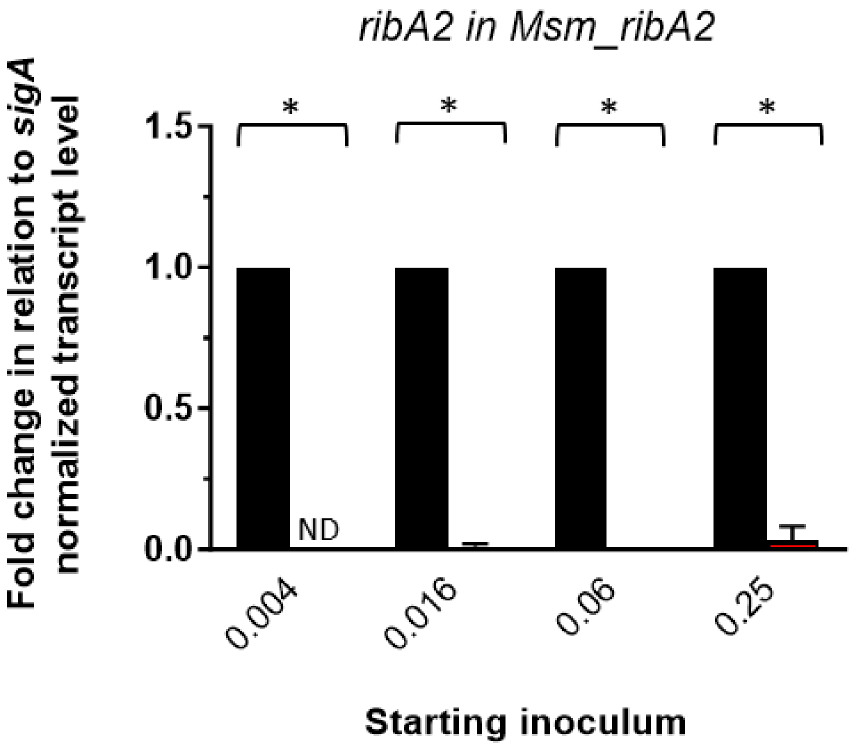

FIG. S7. Efficacy of CRISPRi-dependent silencing of *ribA2* in *Msm_ribA2* as a function of inoculum size. *Msm_ribA2* was inoculated at a starting OD_{600} value of 0.004, 0.016, 0.06 or 0.25, grown in the presence or absence of ATc and harvested after 24 hours. Fold change in expression of *ribA2* in the presence of ATc (red bars) was compared to that in the absence of ATc (black bars). Error bars represent the SD derived from three biological replicates. Statistical comparisons were performed using a two-way ANOVA and Sidak's multiple comparison test whereby statistical significance is represented by $p < 0.05$, denoted by an asterisk. ND – not detected, RNA extracted was below the level of detection.

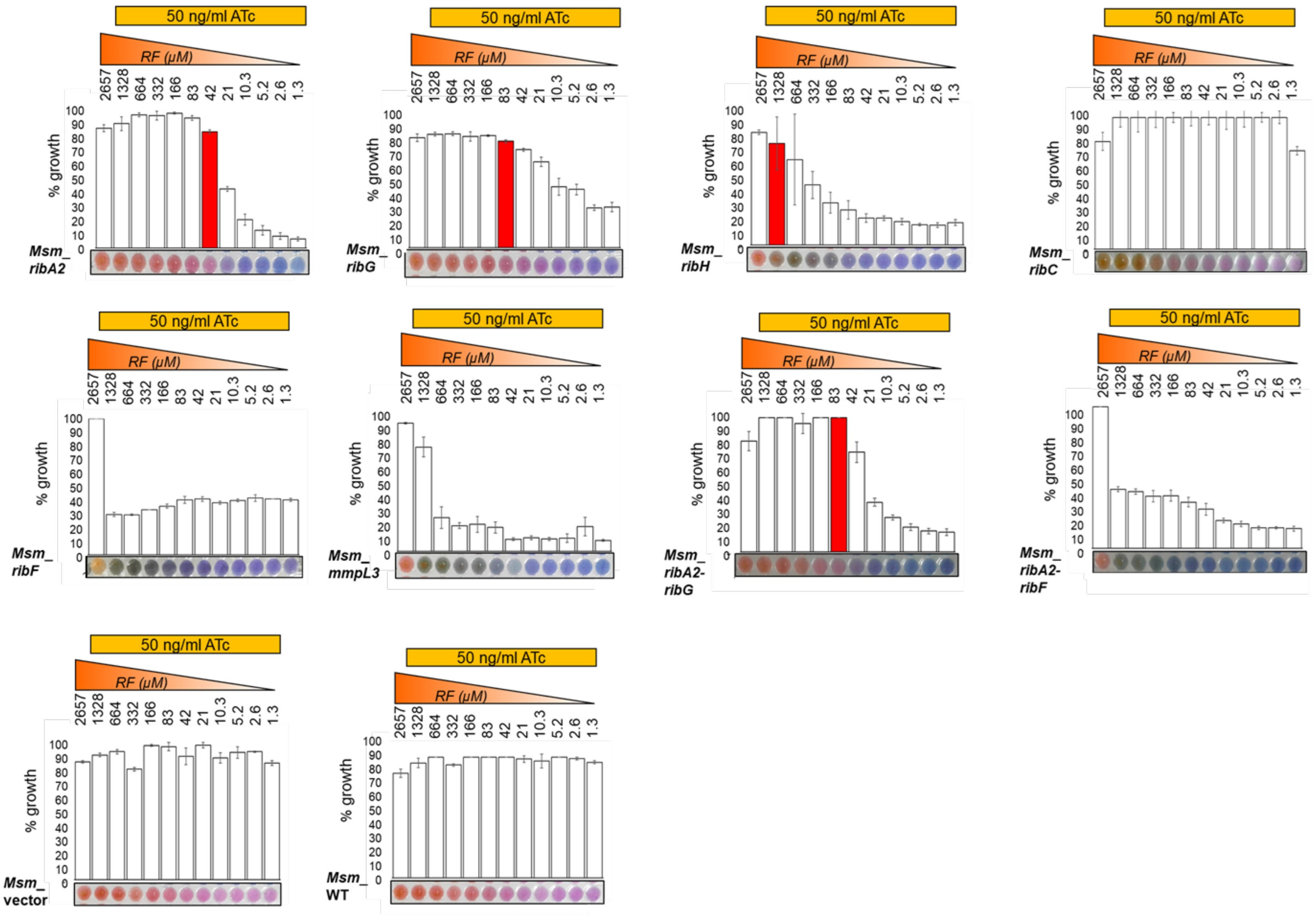

FIG. S8. Impact of exogenous RF on growth of Msm RF pathway hypomorphs. The RF dose-responsiveness of growth was determined using a microbroth dilution alamar blue (MABA) colorimetric assay at a fixed concentration of ATc (50 ng/ml) and a RF gradient (1.3 µM – 2.7 mM). The minimum concentration of RF required to restore ≥80% of wildtype growth is shown in red. Error bars represent the SD derived from three technical replicates.

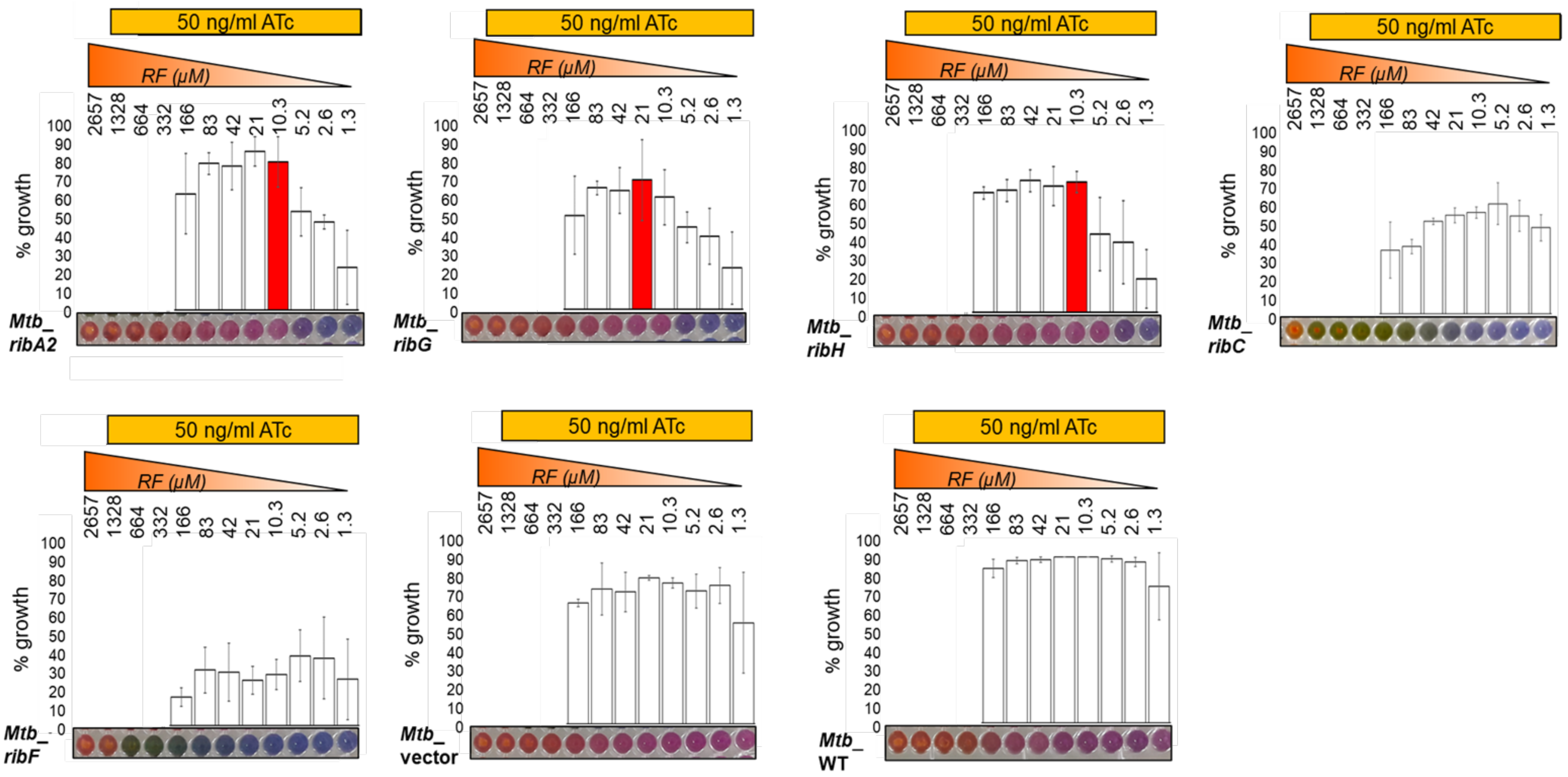

FIG. S9. Impact of exogenous RF on growth of Mtb RF pathway hypomorphs. The RF dose-responsiveness of growth was determined using a microbroth dilution alamar blue (MABA) colorimetric assay at a fixed concentration of ATc (50 ng/ml) and a RF gradient (1.3 µM – 2.7 mM). The minimum concentration of RF required to restore growth to ≥80% of the wildtype is shown in red. Error bars represent the SD derived from three technical replicates. Values from the first four wells have been excluded owing to the dense RF pellet obscuring the spectrophotometric reading.

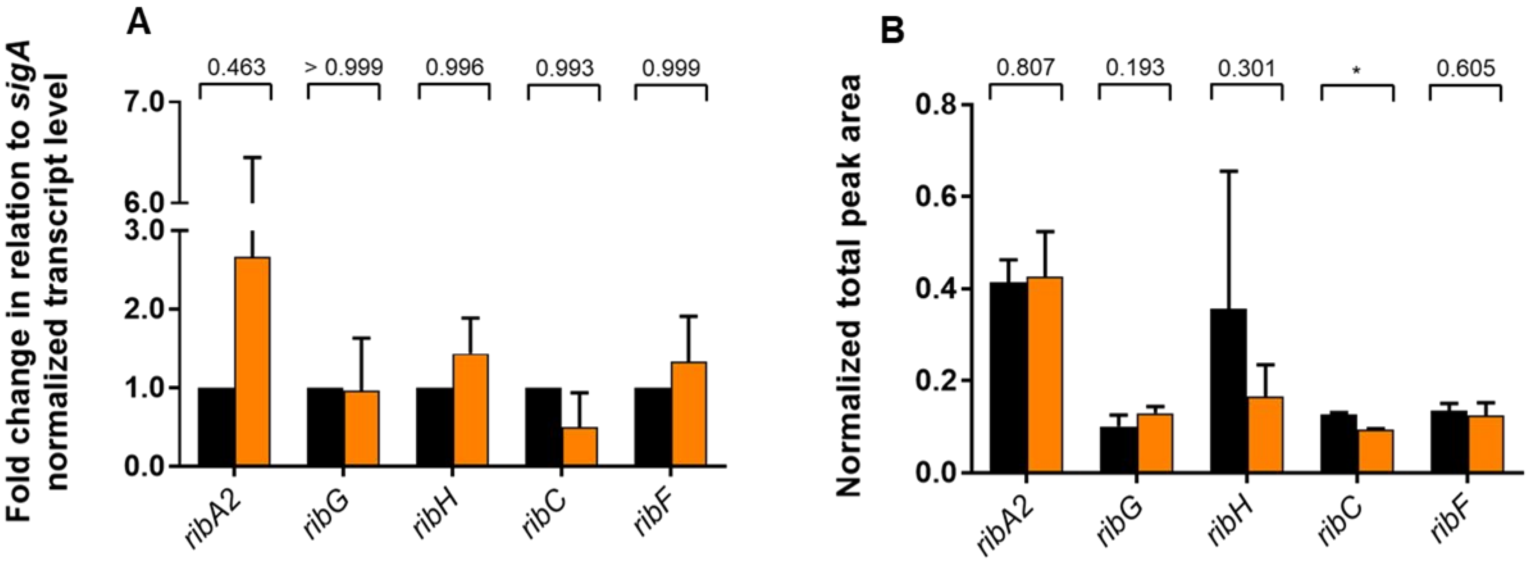

FIG. S10. Impact of an exogenous supply of RF on expression of RF pathway genes and abundance of pathway proteins in wildtype Msm. (**A**) The strain was inoculated at $1.5x10^5$-$3x10^5$ CFU/ml and either grown in standard 7H9 media or 7H9 media supplemented with 83 µM RF for 24 hrs prior to harvesting. Fold gene expression in the absence (black) or presence of exogenous RF (orange). Error bars represent SD derived from three biological replicates. Statistical comparisons were performed using a two-way ANOVA and Sidak's multiple comparison test whereby statistical significance is represented by $p < 0.05$, denoted by an asterisk. (**B**) Relative protein abundance determined by MRM-MS (B) in the absence (black) or presence of RF supplement (orange). Error bars represent the SD derived from three biological replicates. Statistical comparisons were performed by unpaired Student's t-test. Statistical significance is represented by $p<0.05$ and denoted with an asterisk.

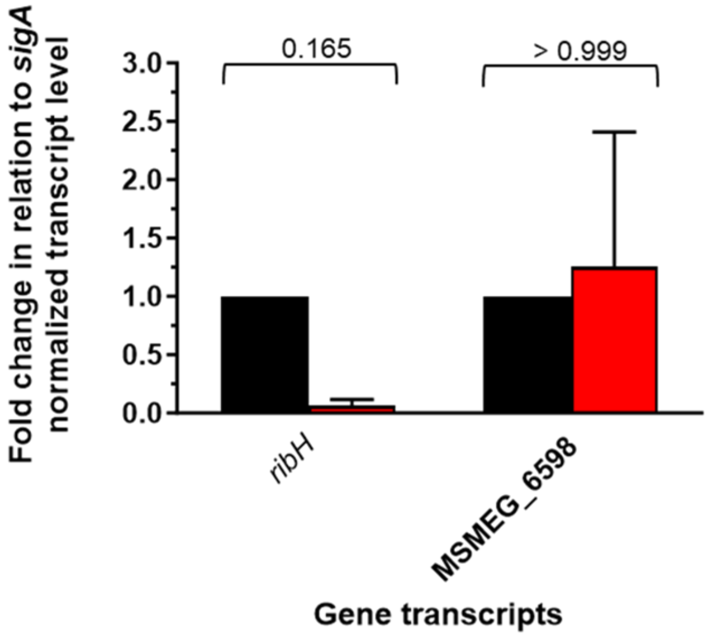

Fig. S11. Impact of gene silencing of *ribH* on the expression of MSMEG_6598 in Msm_*ribH*. Fold gene expression in the presence of ATc (red bars) compared to the absence of ATc (black bars). Error bars represent the SD derived from three biological replicates. Statistical comparisons were performed using a two-way ANOVA and either the Sidak's or Dunnett's multiple comparison test whereby statistical significance is represented by $p < 0.05$, denoted by an asterisk.

TABLE S1. Genes involved in RF biosynthesis and utilization in Mtb and Msm

**Gene**	**Mtb name (H37Rv)**	**Msm name ($mc^2$155)**	**Gene product**	***In vitro* essentiality in Mtb**	**Vulnerability index (1)[a]**
ribA2	Rv1415	MSMEG_3072 (*ribAB*)	GTP cyclohydrolase II + 3,4-dihydroxy-2-butanone 4-phosphate synthase (DHBP synthase)	Essential (2-4). Non-essential (5)[b]	-11.8510 (Mtb) -6.9540 (Msm)
ribA1	Rv1940	MSMEG_3471	GTP cyclohydrolase II	Non-essential (2-5)	1.0210 (Mtb) 0.8120 (Msm)
ribG	Rv1409	MSMEG_3067 (*ribD*)	Riboflavin-specific deaminase + 5-amino-6-(5-phosphoribosylamino) uracil reductase	Essential (2-4) Non-essential (5)	-4.8530 (Mtb) -0.2550 (Msm)
ribH	Rv1416	MSMEG_3073 MSMEG_6598 (*ribE*)	Probable riboflavin synthase beta chain (lumazine synthase)	Essential (2-4)	-6.4400 (Mtb) -1.9430 (Msm) 0.2170 (Msm)
ribC	Rv1412	MSMEG_3071 (*ribE*)	Probable riboflavin synthase alpha chain	Non-essential (2-4)	-5.1780 (Mtb) -1.0910 (Msm)
ribF	Rv2686c	MSMEG_2653 (*ribF*)	Flavokinase + FAD synthetase	Essential (2-5)	-9.8770 (Mtb) -5.4490 (Msm)

a. Taken from https://pebble.rockefeller.edu/
b. From Minato *et al.* (ref. 5), essentiality assessed in the presence of riboflavin supplement.

TABLE S2. sgRNAs synthesized to create Msm RF pathway hypomorphs.

Primer name	Primer sequence (5'-3')	Targeted gene
MSMEG3072_CrispF1	GGGAGTCCTGGTTCACCGCGTACA	MSMEG_ 3072 (*ribA2*)
MSMEG3072_CrispR1	AAACTGTACGCGGTGAACCAGGAC	
MSMEG3072_CrispF2	GGGAGCCACCGTCCTTGGCGCGCA	
MSMEG3072_CrispR2	AAACTGCGCGCCAAGGACGGTGGC	
MSMEG3072_CrispF3	GGGAGTCGACGGCGGCCTCGGTGTGG	
MSMEG3072_CrispR3	AAACCCACACCGAGGCCGCCGTCGAC	
MSMEG3072_CrispF4	GGGAGCGGAACTCGCCGTGCCGCG	
MSMEG3072_CrispR4	AAACCGCGGCACGGCGAGTTCCGC	
MSMEG3072_CrispF5	GGGAGAACCGAAGACGTCACCCGT	
MSMEG3072_CrispR5	AAACACGGGTGACGTCTTCGGTTC	
MSMEG3072_CrispF6	GGGAGCCCTCGTGTCCGCGCATGT	
MSMEG3072_CrispR6	AAACACATGCGCGGACACGAGGGC	
MSMEG3072_CrispF7	GGGAGCCGTAGTCGCGGGCGTCGG	
MSMEG3072_CrispR7	AAACCCGACGCCCGCGACTACGGC	
MSMEG3072_CrispF8	GGGAGCCGATGCCGTAGTCGCGGGCG	
MSMEG3072_CrispR8	AAACCGCCCGCGACTACGGCATCGGC	
MSMEG3072_CrispF9	GGGAGTTCTCCTTGTTCGCCCGCACCGGCA	
MSMEG3072_CrispF9	AAACTGCCGGTGCGGGCGAACAAGGAGAAC	
MSMEG3072_CrispF10	GGGAGTTCTCCTTGTTCGCCCGCA	
MSMEG3072_CrispR10	AAACTGCGGGCGAACAAGGAGAAC	
MSMEG3072_CrispF11	GGGAATATCGGCTATCGCCCTCTCG	
MSMEG3072_CrispR11	AAACCGAGAGGGCGATAGCCGATAT	
MSMEG3072_CrispF12	GGGAATGCTCTTGTAGCCCACCGC	
MSMEG3072_CrispR12	AAACGCGGTGGGCTACAAGAGCAT	
MSMEG3072_CrispF13	GGGAATCGACCGGATCCCGAGGTCCA	
MSMEG3072_CrispR13	AAACTGGACCTCGGGATCCGGTCGAT	
MSMEG3072_CrispF14	GGGAGCGCTTGGCCGGGTTGTTGG	
MSMEG3072_CrispR14	AAACCCAACAACCCGGCCAAGCGC	
MSMEG3072_CrispF15	GGGAATTCCGTCGGCCTGCGGTCAC	
MSMEG3072_CrispR15	AAACGTGACCGCAGGCCGACGGAAT	
MSMEG3067_CrispF1	GGGAGCCCGCGGCCACCGGGTTGGGG	MSMEG_ 3067 (*ribG*)
MSMEG3067_CrispR1	AAACCCCCAACCCGGTGGCCGCGGGC	
MSMEG3067_CrispF2	GGGAGTCGCGAATTTCCATGTGACAT	
MSMEG3067_CrispR2	AAACATGTCACATGGAAATTCGCGAC	
MSMEG3067_CrispF3	GGGAACGGTCGGCGAGGGTGCCGT	
MSMEG3067_CrispR3	AAACACGGCACCCTCGCCGACCGT	
MSMEG3067_CrispF4	GGGAGTCGTTGAGCACGTTGGCATC	
MSMEG3067_CrispR4	AAACGATGCCAACGTGCTCAACGAC	
MSMEG3067_CrispF5	GGGAGCGAATCGTCGTTGAGCACG	
MSMEG3067_CrispR5	AAACCGTGCTCAACGACGATTCGC	
MSMEG3067_CrispF6	GGGAACCATGGTGCGCGAATCGTCGT	
MSMEG3067_CrispR6	AAACACGACGATTCGCGCACCATGGT	
MSMEG3067_CrispF7	GGGAGGTGCGATCCGACAGCGCAC	
MSMEG3067_CrispR7	AAACGTGCGCTGTCGGATCGCACC	
MSMEG3067_CrispF8	GGGAGCGGTTCACCACGCCCGCGC	
MSMEG3067_CrispR8	AAACGCGCGGGCGTGGTGAACCGC	
MSMEG3067_CrispF9	GGGAGACCGCGGTGATCGGTCCCC	
MSMEG3067_CrispF9	AAACGGGGACCGATCACCGCGGTC	

MSMEG3067_CrispF10	GGGAACCGCTGCGCGTGCGCGATGC	
MSMEG3067_CrispR10	AAACGCATCGCGCACGCGCAGCGGT	
MSMEG3067_CrispF11	GGGAATCGAGGATCACCGCTCCGACA	
MSMEG3067_CrispR11	AAACTGTCGGAGCGGTGATCCTCGAT	
MSMEG3067_CrispF12	GGGAGCCTGCGACCTGGCCGTCGCGAT	
MSMEG3067_CrispR12	AAACATCGCGACGGCCAGGTCGCAGGC	
MSMEG3067_CrispF13	GGGAACCCGAGGTGACCTCGATGCC	
MSMEG3067_CrispR13	AAACGGCATCGAGGTCACCTCGGGT	
MSMEG3073_CrispF1	GGGAACGCGTCGATCTCGGGCAGAT	MSMEG_ 3073 (*ribH*)
MSMEG3073_CrispR1	AAACATCTGCCCGAGATCGACGCGT	
MSMEG3073_CrispF2	GGGAGTCAGCGACGACGCGTCGATCT	
MSMEG3073_CrispR2	AAACAGATCGACGCGTCGTCGCTGAC	
MSMEG3073_CrispF3	GGGAGGCGACCTTGCGCGCGCCCT	
MSMEG3073_CrispR3	AAACAGGGCGCGCGCAAGGTCGCC	
MSMEG3073_CrispF4	GGGAGCACCCGCACCACCGTCGGGT	
MSMEG3073_CrispR4	AAACACCCGACGGTGGTGCGGGTGC	
MSMEG3073_CrispF5	GGGAGACCGGGATCTCGATGGCGC	
MSMEG3073_CrispR5	AAACGCGCCATCGAGATCCCGGTC	
MSMEG3073_CrispF6	GGGAGGCCAGAGCCTGCGCCACGA	
MSMEG3073_CrispR6	AAACTCGTGGCGCAGGCTCTGGCC	
MSMEG3073_CrispF7	GGGAACACGGGTCAGGCCCTGCGTG	
MSMEG3073_CrispR7	AAACCACGCAGGGCCTGACCCGTGT	
MSMEG3073_CrispF8	GGGAGCCTGCTCCTCGGTGTTGGTGG	
MSMEG3073_CrispR8	AAACCCACCAACACCGAGGAGCAGGC	
MSMEG3073_CrispF9	GGGAGCCCTTGTCCTCGGTGGAAC	
MSMEG3073_CrispR9	AAACGTTCCACCGAGGACAAGGGC	
MSMEG3073_CrispF10	GGGAGCCTGCGCGCCCTTGTCCTCG	
MSMEG3073_CrispR10	AAACCGAGGACAAGGGCGCGCAGGC	
MSMEG3071_CrispF1:	GGGAATCTCAAACCAGTCATCACCAATG	MSMEG_ 3071 (*ribC*)
MSMEG3071_CrispR1:	AAACCATTGGTGATGACTGGTTTGAGAT	
MSMEG3071_CrispF2:	GGGAGCGTGGTCAGCTCGCGAGTGG	
MSMEG3071_CrispR2:	AAACCCACTCGCGAGCTGACCACGC	
MSMEG3071_CrispF3:	GGGAATCTCAAACCAGTCATCACCA	
MSMEG3071_CrispR3:	AAACTGGTGATGACTGGTTTGAGAT	
MSMEG3071_CrispF4:	GGGAACCACGTACCGCGAGAGCGCGG	
MSMEG3071_CrispR4:	AAACCCGCGCTCTCGCGGTACGTGGT	
MSMEG3071_CrispF5:	GGGAATCGGCGGTGAACGCGCCGT	
MSMEG3071_CrispR5:	AAACACGGCGCGTTCACCGCCGAT	
MSMEG_2653_CrispF1	GGGAGCCGTCGAACACCCCGATGG	MSMEG_ 2653 (*ribF*)
MSMEG_2653_CrispR1	AAACCCATCGGGGTGTTCGACGGC	
MSMEG_2653_CrispF2	GGGAGCAGGCCCGGATGTAGGTCGA	
MSMEG_2653_CrispR2	AAACTCGACCTACATCCGGGCCTGC	
MSMEG_2653_CrispF3	GGGAGCCTCGACCGTGCGGGTGCGGCC	
MSMEG_2653_CrispR3	AAACGGCCGCACCCGCACGGTCGAGGC	
MSMEG_2653_CrispF4	GGGAGAAGCGCTCACCGGCCTTGC	
MSMEG_2653_CrispR4	AAACGCAAGGCCGGTGAGCGCTTC	
MSMEG_2653_CrispF5	GGGAAGCTGCGCCGGGTGGTTACC	
MSMEG_2653_CrispR5	AAACGGTAACCACCCGGCGCAGCT	
MSMEG_2653_CrispF6	GGGAGTCGGAGGTGAACGGCATCAC	
MSMEG_2653_CrispR6	AAACGTGATGCCGTTCACCTCCGAC	

MSMEG_2653_CrispF7	GGGAGGCGGTGCCACGTTGGCCGT	
MSMEG_2653_CrispR7	AAACACGGCCAACGTGGCACCGCC	
MSMEG_2653_CrispF8	GGGAGTCTTGCCCACGCCAGCGTTGC	
MSMEG_2653_CrispR8	AAACGCAACGCTGGCGTGGGCAAGAC	
MSMEG_2653_CrispF9	GGGAGACGACGTGCAGGTGCTCGA	
MSMEG_2653_CrispR9	AAACTCGAGCACCTGCACGTCGTC	
MSMEG_2653_CrispF10	GGGAGTCGGAGGTGAACGGCATCA	
MSMEG_2653_CrispR10	AAACTGATGCCGTTCACCTCCGAC	
MSMEG_MmpL3_CrispF1	GGGAGCGACAGACTGGCTGCCCTCGTC	MSMEG_ 0250
MSMEG_MmpL3_CrispR1	AAACGACGAGGGCAGCCAGTCTGTCGC	
MSMEG_NT_F1	GGGAGCATCCGGAGCCCGTCCGTTAA	Non-targeting sgRNA
MSMEG_NT_R1	AAACTTAACGGACGGGCTCCGGATGC	

TABLE S3. sgRNAs synthesized to create Mtb RF pathway hypomorphs.

Primer name	Primer sequence (5'-3')	Targeted gene
Rv1415_CrispF1	GGGAGTCGCAGATGGCACCGTCCA	Rv1415 (*ribA2*)
Rv1415_CrispR1	AAACTGGACGGTGCCATCTGCGAC	
Rv1415_CrispF2	GGGAACCCCACCATCCTTGGCCCGCA	
Rv1415_CrispR2	AAACTGCGGGCCAAGGATGGTGGGGT	
Rv1415_CrispF3	GGGAGCACTCCGAATGCACCCGGA	
Rv1415_CrispR3	AAACTCCGGGTGCATTCGGAGTGC	
Rv1415_CrispF4	GGGAGACCCAAACACATCGCCGGT	
Rv1415_CrispR4	AAACACCGGCGATGTGTTTGGGTC	
Rv1415_CrispF5	GGGAGCCCTCGTGGCCACGCATGT	
Rv1415_CrispR5	AAACACATGCGTGGCCACGAGGGC	
Rv1415_CrispF6	GGGAGCCGATCCCGTAATCCCTTGCG	
Rv1415_CrispR6	AAACCGCAAGGGATTACGGGATCGGC	
Rv1415_CrispF7	GGGAATCGAACGTACCCCAAGATCGA	
Rv1415_CrispR7	AAACTCGATCTTGGGGTACGTTCGAT	
Rv1415_CrispF8	GGGAACCCGCTTGGCCGGGTTGTTGG	
Rv1415_CrispR8	AAACCCAACAACCCGGCCAAGCGGGT	
Rv1415_CrispF9	GGGAGTTCTCCGCGTTGGCCCGCACCGGCA	
Rv1415_CrispR9	AAACTGCCGGTGCGGGCCAACGCGGAGAAC	
Rv1415_CrispF10	GGGAACAAGGCACCGCCGAATTCTC	
Rv1415_CrispR10	AAACGAGAATTCGGCGGTGCCTTGT	
Rv1415_CrispF12:	GGGAGCCTCGGTGTGGCCGGGCCGGC	
Rv1415_CrispR12:	AAACGCCGGCCCGGCCACACCGAGGC	
Rv1415_CrispF13:	GGGAGCTTGCGCCGCCATTCGATCAAG	
Rv1415_CrispR13:	AAACCTTGATCGAATGGCGGCGCAAGC	
Rv1415_CrispF14:	GGGAGCGAAACTCCCCATGACGAG	
Rv1415_CrispR14:	AAACCTCGTCATGGGGAGTTTCGC	
Rv1409_CrispR1	AAACCCGACGACCCGGCCCTGACC	Rv1409 (*ribG*)
Rv1409_CrispF2	GGGAATCATGGTGCGTGCCTCGTCGT	
Rv1409_CrispR2	AAACACGACGAGGCACGCACCATGAT	
Rv1409_CrispF3	GGGAGGTGCGATCCGACAACGCCC	
Rv1409_CrispR3	AAACGGGCGTTGTCGGATCGCACC	
Rv1409_CrispF4	GGGAGAGGGTGGGACCTCCTTCCA	
Rv1409_CrispR4	AAACTGGAAGGAGGTCCCACCCTC	
Rv1409_CrispF5	GGGAGGCGAGGGTGGGACCTCCTT	
Rv1409_CrispR5	AAACAAGGAGGTCCCACCCTCGCC	
Rv1409_CrispF6	GGGAATCCGGTTGATCGCACCCGCTC	
Rv1409_CrispR6	AAACGAGCGGGTGCGATCAACCGGAT	
Rv1409_CrispF7	GGGAACCGCGGTAACCGGACCGCCCA	
Rv1409_CrispR7	AAACTGGGCGGTCCGGTTACCGCGGT	
Rv1409_CrispF8	GGGAGCCCGGCGCAGCGCCACCACC	
Rv1409_CrispR8	AAACGGTGGTGGCGCTGCGCCGGGC	
Rv1409_CrispF9	GGGAATGGTGACCACCACGATGGCG	
Rv1409_CrispR9	AAACCGCCATCGTGGTGGTCACCAT	
Rv1409_CrispF10	GGGAGGCGTACTTCCAGGTGACAT	
Rv1409_CrispR10	AAACATGTCACCTGGAAGTACGCC	
Rv1409_CrispF11:	GGGAATCCGGTTGATCGCACCCGCTCG	
Rv1409_CrispR11:	AAACCGAGCGGGTGCGATCAACCGGAT	
Rv1409_CrispF12:	GGGAATAAGTCGTGCCTTTGACCTG	

Rv1409_CrispR12:	AAACCAGGTCAAAGGCACGACTTAT	
Rv1409_CrispF13:	GGGAGCCGGCGCCGACGATCCGACCG	
Rv1409_CrispR13:	AAACCGGTCGGATCGTCGGCGCCGGC	
Rv1416_CrispF1:	GGGAACGCATCCAGCGACGGCAGAT	Rv1416 (*ribH*)
Rv1416_CrispR1:	AAACATCTGCCGTCGCTGGATGCGT	
Rv1416_CrispF2:	GGGAGCACACCAGACGCATCCAGCG	
Rv1416_CrispR2:	AAACCGCTGGATGCGTCTGGTGTGC	
Rv1416_CrispF3:	GGGAACCACCGGAATCTCGATCGCGC	
Rv1416_CrispR3:	AAACGCGCGATCGAGATTCCGGTGGT	
Rv1416_CrispF4:	GGGAGGCCAATTCCTGCGCCACCA	
Rv1416_CrispR4:	AAACTGGTGGCGCAGGAATTGGCC	
Rv1416_CrispF5:	GGGAATCACGACGCCAAGTGCGACG	
Rv1416_CrispR5:	AAACCGTCGCACTTGGCGTCGTGAT	
Rv1412_CrispF1:	GGGAGCGCCGAGCCCGGAGACCGTC	Rv1412 (*ribC*)
Rv1412_CrispR1:	AAACGACGGTCTCCGGGCTCGGCGC	
Rv1412_CrispF2:	GGGAGCGCCGAGCCCGGAGACCGTC	
Rv1412_CrispR2:	AAACGACGGTCTCCGGGCTCGGCGC	
Rv1412_CrispF3:	GGGAGCGTGGTCAGCTCCCGGGTCG	
Rv1412_CrispR3:	AAACCGACCCGGGAGCTGACCACGC	
Rv1412_CrispF4:	GGGAGCACCACTTCCCAGTGCTCG	
Rv1412_CrispR4:	AAACCGAGCACTGGGAAGTGGTGC	
Rv1412_CrispF5:	GGGAGTCGGCGGTGAATTGGCCGT	
Rv1412_CrispR5:	AAACACGGCCAATTCACCGCCGAC	
Rv2786c_CrispF1	GGGAGCTTCGACGGTGCGGGTGCGTCC	Rv2786c (*ribF*)
Rv2786c_CrispR1	AAACGGACGCACCCGCACCGTCGAAGC	
Rv2786c_CrispF2	GGGAGCAGGACCGGATGTAGGTGGA	
Rv2786c_CrispR2	AAACTCCACCTACATCCGGTCCTGC	
Rv2786c_CrispF3	GGGAATCGGCGGCGCCACGTTCGCGG	
Rv2786c_CrispR3	AAACCCGCGAACGTGGCGCCGCCGAT	
Rv2786c_CrispF4	GGGAACGCAGGACCGGATGTAGGT	
Rv2786c_CrispR4	AAACACCTACATCCGGTCCTGCGT	
Rv2786c_CrispF5	GGGAATCGGTGGTGAACGGCATCAC	
Rv2786c_CrispR5	AAACGTGATGCCGTTCACCACCGAT	
Rv2786c_CrispF6	GGGAAGCGGCGTACACGCCGTCGG	
Rv2786c_CrispR6	AAACCCGACGGCGTGTACGCCGCT	
Rv2786c_CrispF7	GGGAACCTCCACCACATGTAGGTGCTCGA	
Rv2786c_CrispR7	AAACTCGAGCACCTACATGTGGTGGAGGT	
Rv2786c_CrispF8	GGGAATCGGTGGTGAACGGCATCA	
Rv2786c_CrispR8	AAACTGATGCCGTTCACCACCGAT	
Rv2786c_CrispF9	GGGAACCATGTCGCCGGCGTCCAC	
Rv2786c_CrispR9	AAACGTGGACGCCGGCGACATGGT	
Rv2786c_CrispF10	GGGAGTGCGGGTGCGTCCGGAGAAG	
Rv2786c_CrispR10	AAACCTTCTCCGGACGCACCCGCAC	
Mtb_MmpL3_CrispF1:	GGGAACCCGGTTAACCAGCTTGCC	Rv0206c (*mmpL3*)
Mtb_MmpL3_CrispR1:	AAACGGCAAGCTGGTTAACCGGGT	

TABLE S4. ORBIT oligo to create targeted knockout in Msm *ribH*.

Primer name	Primer	Application
MSMRibH_ORB_KO	TGCACGTCCCAGGTCTCGTCCGCCATCTGTGA GGCCATCGTCACGCGCGGCGCAATTCGCGCAG CGTCACGGTTTGTCTGGTCAACCACCGCGGTC TCAGTGGTGTACGGTACAAACCGGGCAGATCG GGGACGCCGACGCCGCTCACTGCGCCCCGCCC GATTCCGTCGGCCTGCGGTCACCCAGCA	Msm *ribH* knockout

TABLE S5. Plasmids used in this study.

Plasmid	Description	Reference
pLJR962	ATc-inducible (P_{tet}) *Streptococcus thermophilius* (Sth) dCas9 and P_{tet} sgRNA, integrating at the L5 locus in *Msm*; Kan^R	(6)
pLJR965	ATc-inducible (P_{tet}) Sth dCas9 and P_{tet} sgRNA, integrating at the L5 locus in *Mtb*; Kan^R	(6)
pKM461	Plasmid expresses P_{tet} Che9c phage RecT annealase and P_{tet} Bxb1 phage integrase; *oriE*, *oriM*, *sacB* ; Kan^R, Tet^R	(7)
pKM464	Non-replicating vector carrying *attB*; Hyg^R	(7)
pLJR962_*ribA2*	Derivative of pLJR962 carrying a *ribA2* (MSMEG_3072) targeting sgRNA; Kan^R	This study
pLJR962_*ribG*	Derivative of pLJR962 carrying a *ribG* (MSMEG_3067) targeting sgRNA; Kan^R	This study
pLJR962_*ribH*	Derivative of pLJR962 carrying a *ribH* (MSMEG_3073) targeting sgRNA; Kan^R	This study
pLJR962_*ribC*	Derivative of pLJR962 carrying a *ribC* (MSMEG_3071) targeting sgRNA; Kan^R	This study
pLJR962_*ribF*	Derivative of pLJR962 carrying a *ribF* (MSMEG_2653) targeting sgRNA; Kan^R	This study
pLJR962_*ribA2_ribG*	Derivative of pLJR962 carrying *ribA2* (MSMEG_3072) and *ribG* (MSMEG_3067) targeting sgRNAs; Kan^R	This study
pLJR962_*ribA2_ribF*	Derivative of pLJR962 carrying a *ribA2* (MSMEG_3072) and a *ribF* (MSMEG_2653) targeting sgRNAs; Kan^R	This study
pLJR962_*mmpL3*	Derivative of pLJR962 carrying a *mmpL3* (MSMEG_0250) targeting sgRNA; Kan^R	This study
pLJR962_NT	Derivative of pLJR962 carrying a non-targeting sgRNA; Kan^R	This study
pLJR962_6598	Derivative of pLJR962 carrying a MSMEG_6598 targeting sgRNA; Kan^R	This study
pLJR965_*ribA2*	Derivative of pLJR965 carrying a *ribA2* (Rv1415) targeting sgRNA	This study
pLJR965_*ribG*	Derivative of pLJR965 carrying a *ribG* (Rv1409) targeting sgRNA	This study
pLJR965_*ribH*	Derivative of pLJR965 carrying a *ribH* (Rv1416) targeting sgRNA	This study
pLJR965_*ribC*	Derivative of pLJR965 carrying a *ribC* (Rv1412) targeting sgRNA	This study

pLJR965_*ribF*	Derivative of pLJR965 carrying a *ribF* (Rv2786c) targeting sgRNA	This study
pLJR965_*mmpL3*	Derivative of pLJR965 carrying a *mmpL3* (Rv0206c) targeting sgRNA	This study

TABLE S6. Strains used in this study.

Strain	Description	Reference
Escherichia coli DH5α	F^- φ80*lac*ZΔM15 Δ(*lac*ZYA-*arg*F)U169 *rec*A1 *end*A1 *hsd*R17(r_K^-, m_K^+) *pho*A *sup*E44 λ^- *thiA*-1 *gyr*A96 *rel*A1	New England Biolabs
mc^{2}155	High frequency transformation mutant of *M. smegmatis* mc^{2}6 ATCC®™	(8)
Msm_ribA2	Anhydrotetracycline (ATc)-inducible Msm hypomorph targeting *ribA2* generated using plasmid pLJR962_*ribA2*	This study
Msm_ribG	ATc-inducible Msm hypomorph targeting *ribG* generated using plasmid pLJR962_*ribG*	This study
Msm_ribH	ATc-inducible Msm hypomorph targeting *ribH* generated using plasmid pLJR962_*ribH*	This study
Msm_ribC	ATc-inducible Msm hypomorph targeting *ribC* generated using plasmid pLJR962_*ribC*	This study
Msm_ribF	ATc-inducible Msm hypomorph targeting *ribF* generated using plasmid pLJR962_*ribF*	This study
Msm_ribA2_ribG	ATc-inducible Msm hypomorph targeting *ribA2* and *ribG* generated using plasmid pLJR962_*ribA2_ribG*	This study
Msm_ribA2_ribF	ATc-inducible Msm hypomorph targeting *ribA2* and *ribF* generated using plasmid pLJR962_*ribA2_ribF*	This study
Msm_mmpL3	ATc-inducible Msm hypomorph targeting *mmpL3* generated using plasmid pLJR962_*mmpL3*	This study
*Msm*_NT	ATc-inducible Msm control strain generated using pLJR962 and a non-targeting sgRNA	This study
*Msm*_vector	ATc-inducible Msm control strain generated using pLJR962 and no targeting sgRNA	This study
*Msm*_MSMEG_6598	ATc-inducible Msm hypomorph targeting MSMEG_6598 generated using plasmid pLJR962_ MSMEG_6598	This study
Msm ΔribH	In-frame deletion mutant lacking internal region in *ribH* generated using construct pKM464 and directed oligo; Hyg^R marked mutant generated using ORBIT. This strain is not a RF auxotroph.	This study
Msm ΔribH MSMEG_6598	ATc-inducible Msm hypomorph targeting MSMEG_6598 generated using plasmid pLJR962_ MSMEG_6598 in *Msm ΔribH*	This study
H37RvMA	Mtb H37Rv isolate ATCC® 27294™ virulent laboratory strain	(9)
Mtb_ribA2	ATc-inducible Mtb hypomorph targeting *ribA2* generated using plasmid pLJR965_*ribA2*	This study
Mtb_ribG	ATc-inducible Mtb hypomorph targeting *ribG* generated using plasmid pLJR965_*ribG*	This study
Mtb_ribH	ATc-inducible Mtb hypomorph targeting *ribH* generated using plasmid pLJR965_*ribH*	This study
Mtb_ribC	ATc-inducible Mtb hypomorph targeting *ribC* generated using plasmid pLJR965_*ribC*	This study
Mtb_ribF	ATc-inducible Mtb hypomorph targeting *ribF* generated using plasmid pLJR965_*ribF*	This study

*Mtb*_vector	ATc-inducible Mtb strain carrying pLJR965 and no targeting sgRNA	This study
Mtb_mmpL3	ATc-inducible Mtb strain carrying pLJR965 targeting *mmpL3* generated using plasmid pLJR965 *mmpL3*	This study

TABLE S7. Primers used for qRT-PCR analyses.

**Primer name**	**Primer sequence (5’-3’)**	**Amplicon size (bp)**	**Gene region amplified**
qRT_MSM_3072Fwd	GCCGACGATTTCACCAAGC	159	MSMEG_3072 (*ribA2*)
qRT_MSM_3072Rev	GTCCTTCTGGCTGACGATCTC		
qRT_MSM_3067Fwd	TCAACCGTTGCGTGTCGT	151	MSMEG_3067 (*ribG*)
qRT_MSM_3067Rev	GCCCTCGAGCATGATGTC		
qRT_MSM_3073Fwd	CACCGAGATCTGCGACGC	102	MSMEG_3073 (*ribH*)
qRT_MSM_3073Rev	TGTCTCACCTTGGATCACGAC		
qRT_MSM_3071Fwd	TTGGTGATGACTGGTTTGAGA	106	MSMEG_3071 (*ribC*)
qRT_MSM_3071Rev	ATGACGTCCACTTCCAGGTTC		
qRT_MSM_2653Fwd	GATCGACGTGTTCCTGGTGAT	163	MSMEG_2653 (*ribF*)
qRT_MSM_2653Rev	CAGCATGTCGACGTTGCC		
qRT_MSM_0250Fwd	AACGATCCGGAGAAGATGTGG	128	MSMEG_0250 (*mmpL3*)
qRT_MSM_0250Rev	CGCAACTCGTCGATCTTCTTG		
qRT_MSM_2758Fwd	ACCAAGGGCTACAAGTTCTCG	198	MSMEG_2758 (*sigA*)
qRT_MSM_2758Rev	CATCTCCTTGGCGAGCTCTTC		
qRT_Rv1415Fwd	CACGGAATGGCATTGGAACTG	144	Rv1415 (*ribA2*)
qRT_Rv1415Rev	GCAGAACCCCACCATCCTTG		
qRT_Rv1409Fwd	GAGGCACGCACCATGATGATC	146	Rv1409 (*ribG*)
qRT_Rv1409Rev	TAGGCCAGGATCCGGTTGA		
qRT_Rv1416Fwd	AATTGGCCCGCAATCATGATG	130	Rv1416 (*ribH*)
qRT_Rv1416Rev	GGCGTCGAGGAATCCAGC		
qRT_Rv1412Fwd	ACATCGTGCAGGGACATGTG	123	Rv1412 (*ribC*)
qRT_Rv1412Rev	AGCCCTTTTCGACGACATAGC		
qRT_Rv2786Fwd	GCTTGGTTCACGGTGCTC	103	Rv2786c (*ribF*)
qRT_Rv2786Rev	CGGAGAAGGTGGGATTGGTC		
qRT_Rv0206Fwd	GACCACCACGATCGTCTTGAT	129	Rv0206c (*mmpL3*)
qRT_Rv0206Rev	TGTCCGTCGACGAATATCCAC		
qRT_Rv2703Fwd	CGGTGATTTCGTCTGGGATGA	113	Rv2703 (*sigA*)
qRT_Rv2703Rev	TGCCGATCTGTTTGAGGTAGG		

TABLE S8. Primers used to amplify junctions between adjacent genes to interrogate operonic structure.

**Primer name**	**Primer sequence (5'-3')**	**Amplicon size (bp)**	**Region amplified**
Msm_ribC_ribA2Fwd	GGGTACGGGTCGGTAAGAG	166	Junction between *ribC* and *ribA2* in Msm
Msm_ribC_ribA2Rev	CGCCCTCTCGACGGAATC		
Msm_ribA2_ribHFwd	CGGTGAGCATGGACGACT	110	Junction between *ribA2* and *ribH* in Msm
Msm_ribA2_ribHRev	TCTCGGGCAGATCGGGGA		
Mtb_ribC_Rv1413Fwd	CGTAGTCGCAAAGTATGTTGAGC	331	Junction between *ribC* and Rv1413 in Mtb
Mtb_ribC_Rv1413Rev	CAATGGGTAGGTGATGAATACGT		
Mtb_Rv1413_Rv1414Fwd	CTTCCCGATCAGCTGCCG	155	Junction between Rv1413 and Rv1414 in Mtb
Mtb_Rv1413_Rv1414Rev	AACGGTCAGCGCGATGTC		
Mtb_Rv1414_ribA2Fwd	GCAACCCTGATTGATCGCTG	170	Junction between Rv1414 and *ribA2* in Mtb
Mtb_Rv1414_ribA2Rev	GTCCAACCTCGTCATCTTTGC		
Mtb_ribA2_ribHFwd	GACAAATTGGGGCACGACTTG	172	Junction between *ribA2* and *ribH* in Mtb
Mtb_ribA2_ribHRev	ATCTTTCCGTGCCAGCTG		

TABLE S9. Peptides used for targeted proteomics by MRM-MS

Protein[¥]	Peptide (m/z)	Transitions	Retention time (min)	LOD (nM)	LOQ (nM)
RibA2	R.LGLLPMYAVNQD**K**.H<br>(731.3921 ++)	P [y9] - 1065.5034+<br>Y [y7] - 837.4101+<br>A [y6] - 674.3468+<br>V [y5] - 603.3097+<br>N [y4] - 504.2413+	7.2	45.97	139.3
	R.GEISGPGSDGDDVLV**R**.V*<br>(786.8786++)	P [y11] - 1129.5484+<br>S [y9] - 975.4742+	5.4	6.35	19.24

		D [y8] - 888.4421+<br>G [y7] - 773.4152+<br>D [y5] - 601.3668+			
RibG	R.VVYAVADPNPVAAGGSA**R**.M*<br>(857.4496++)	P [y11] - 996.5221+<br>P [y9] - 785.4264+<br>A [y6] - 518.2681+<br>G [y5] - 447.2310+<br>G [y4] - 390.2096+	5.4	7.81	23.67
	R.EVSSDANVLNDDS**R**.T	A [y9] - 1003.4803+	6.4	4.03	12.22

	(760.8448 ++)	N [y8] - 932.4432+			
		V [y7] - 818.4003+			
		L [y6] - 719.3319+			
		N [y5] - 606.2478+			
RibH	K.VAADAGIPDPTVV**R**.V	D [y11] - 1139.6055+	5.7	5.4	16.38
	(690.8777++)	A [y10] - 1024.5786+			
		G [y9] - 953.5415+			
		I [y8] - 896.5200+			
		P [y7] - 783.4359			

	R.VLGAIEIPVVAQALA**R**.T<br><br>(810.4958++)	I [y12] - 1279.7732+<br><br>E [y11] - 1166.6892+<br><br>I [y10] - 1037.6466+<br><br>P [y9] - 924.5625+<br><br>V [y7] - 728.4413+	8.7	26.62	80.67
RibC	R.SSLAGVAVGD**R**.V<br><br>(516.2776++)	L [y9] - 857.4839+<br><br>A [y8] - 744.3999+<br><br>G [y7] - 673.3628+<br><br>V [y6] - 616.3413+	4.8	4.49	13.59

		A [y5] - 517.2729+			
	R.IALPPALS**R**.Y*<br><br>(469.2951++)	A [y8] - 824.4989+<br><br>L [y7] - 753.4617+<br><br>P [y6] - 640.3777+<br><br>P [y5] - 543.3249+	6.1	5.42	16.43
RibF	R.YIHELLVEHLHVVEVVVGENFTFG**K**.K<br><br>(969.8516+++)	G [y8] - 450.2165++<br><br>N [y6] - 357.1845++<br><br>F [y5] - 300.1630++<br><br>T [y4] - 226.6288++	N/A	N/A	N/A

		F [y3] - 176.1050++			
	R.NETVTFSSTYI**R**.A<br><br>(l709.3515++)	V [y9] - 1073.5626+<br><br>T [y8] - 974.4942+<br><br>F [y7] - 873.4465+<br><br>S [y6] - 726.3781+	5.9	5.01	15.19

¥Mtb protein names used. RibF peptide 'YIH' was not able to be validated with a labeled synthetic peptide, so it was not included for sample testing.

REFERENCES

1. Bosch B, DeJesus MA, Poulton NC, Zhang W, Engelhart CA, Zaveri A, Lavalette S, Ruecker N, Trujillo C, Wallach JB, Li S, Ehrt S, Chait BT, Schnappinger D, Rock JM. 2021. Genome-wide gene expression tuning reveals diverse vulnerabilities of M. tuberculosis. Cell 184:4579-4592 e24.
2. Sassetti CM, Boyd DH, Rubin EJ. 2003. Genes required for mycobacterial growth defined by high density mutagenesis. Mol Microbiol 48:77-84.
3. Griffin JE, Gawronski JD, Dejesus MA, Ioerger TR, Akerley BJ, Sassetti CM. 2011. High-resolution phenotypic profiling defines genes essential for mycobacterial growth and cholesterol catabolism. PLoS Pathog 7:e1002251.
4. DeJesus MA, Gerrick ER, Xu W, Park SW, Long JE, Boutte CC, Rubin EJ, Schnappinger D, Ehrt S, Fortune SM, Sassetti CM, Ioerger TR. 2017. Comprehensive Essentiality Analysis of the Mycobacterium tuberculosis Genome via Saturating Transposon Mutagenesis. mBio 8.
5. Minato Y, Gohl DM, Thiede JM, Chacon JM, Harcombe WR, Maruyama F, Baughn AD. 2019. Genomewide Assessment of Mycobacterium tuberculosis Conditionally Essential Metabolic Pathways. mSystems 4.
6. Rock JM, Hopkins FF, Chavez A, Diallo M, Chase MR, Gerrick ER, Pritchard JR, Church GM, Rubin EJ, Sassetti CM, Schnappinger D, Fortune SM. 2017. Programmable transcriptional repression in mycobacteria using an orthogonal CRISPR interference platform. Nature Microbiology 2:16274.
7. Murphy KC, Nelson SJ, Nambi S, Papavinasasundaram K, Baer CE, Sassetti CM. 2018. ORBIT: a New Paradigm for Genetic Engineering of Mycobacterial Chromosomes. mBio 9.
8. Snapper SB, Melton RE, Mustafa S, Kieser T, Jacobs WR, Jr. 1990. Isolation and characterization of efficient plasmid transformation mutants of Mycobacterium smegmatis. Mol Microbiol 4:1911-9.
9. Ioerger TR, Feng Y, Ganesula K, Chen X, Dobos KM, Fortune S, Jacobs WR, Jr., Mizrahi V, Parish T, Rubin E, Sassetti C, Sacchettini JC. 2010. Variation among genome sequences of H37Rv strains of Mycobacterium tuberculosis from multiple laboratories. J Bacteriol 192:3645-53.
